# Connectivity of Default-Mode Network Is Associated with Cerebral Edema in Hepatic Encephalopathy

**DOI:** 10.1371/journal.pone.0036986

**Published:** 2012-05-18

**Authors:** Wei-Che Lin, Tun-Wei Hsu, Chao-Long Chen, Changwei W. Wu, Cheng-Hsien Lu, Hsiu-Ling Chen, Shau-Hsuan Li, Pin-Yang Yeh, Yu-Fan Cheng, Ching-Po Lin

**Affiliations:** 1 Department of Diagnostic Radiology, Kaohsiung Chang Gung Memorial Hospital and Chang Gung University College of Medicine, Kaohsiung, Taiwan; 2 Department of Biomedical Imaging and Radiological Sciences, National Yang-Ming University, Taipei, Taiwan; 3 Department of Diagnostic Radiology, Taipei Veterans General Hospital, Taipei, Taiwan; 4 Department of Surgery, Kaohsiung Chang Gung Memorial Hospital and Chang Gung University College of Medicine, Kaohsiung, Taiwan; 5 Institute of Biomedical Engineering, National Central University, Taoyuan, Taiwan; 6 Department of Neurology, Kaohsiung Chang Gung Memorial Hospital and Chang Gung University College of Medicine, Kaohsiung, Taiwan; 7 Department of Internal Medicine, Kaohsiung Chang Gung Memorial Hospital and Chang Gung University College of Medicine, Kaohsiung, Taiwan; 8 Instiute of Allied Health Science, College of Medicine, National Cheng Kung University, Tainan, Taiwan; 9 Department of Psychiatry, Tsyr-Huey Mental Hospital, Kaohsiung, Taiwan; 10 Lab for Brain Connectivity, Institute of Neuroscience, National Yang-Ming University, Taipei, Taiwan; University of California San Francisco, United States of America

## Abstract

Cerebral edema, a well-known feature of acute liver disease, can occur in cirrhotic patients regardless of hepatic encephalopathy (HE) and adversely affect prognosis. This study characterized and correlated functional HE abnormalities in the brain to cerebral edema using resting-state functional magnetic resonance imaging (rs-fMRI) and diffusion tensor imaging (DTI). Forty-one cirrhotic patients (16 without HE, 14 minimal HE, 11 overt HE) and 32 healthy controls were assessed. The HE grade in cirrhotic patients was evaluated by the West Haven criteria and neuro-psychological examinations. Functional connectivity correlation coefficient (fc-CC) of the default mode network (DMN) was determined by rs-fMRI, while the corresponding mean diffusivity (MD) was obtained from DTI. Correlations among inter-cortical fc-CC, DTI indices, Cognitive Ability Screening Instrument scores, and laboratory tests were also analyzed. Results showed that gradual reductions of HE-related consciousness levels, from “without HE” or “minimal HE” to “overt HE”, correlated with decreased anterior-posterior fc-CC in DMN [F(4.415), *p* = 0.000)]. The MD values from regions with anterior-posterior fc-CC differences in DMN revealed significant differences between the overt HE group and other groups. Increased MD in this network was inversely associated with decreased fc-CC in DMN and linearly correlated with poor cognitive performance. In conclusion, cerebral edema can be linked to altered cerebral temporal architecture that modifies both within- and between-network connectivity in HE. Reduced fc-CC in DMN is associated with behavior and consciousness deterioration. Through appropriate targets, rs-fMRI technology may provide relevant supplemental information for monitoring HE and serve as a new biomarker for clinical diagnosis.

## Introduction

Cerebral edema is a well-known feature of acute liver disease that can occur in cirrhotic patients regardless of hepatic encephalopathy (HE) and adversely affect prognosis [1]. There is no effective treatment for HE except liver transplantation. Given the very limited therapeutic efficacy in the overt stage of cerebral edema, awareness of early signs is critical for timely intervention in cirrhotic patients [2]. Currently, diffusion-weighted images can shed light in HE diagnosis by showing that brain water content (mean diffusivity value) increases significantly, progressively affecting more regions as the HE grade increases [3]. However, the efficacy of diffusion techniques in differentiating sub-clinical HE categories remains uncertain.

Astrocyte edema is the phenotype of brain dysmetabolism. It is unlikely that a single mechanism underlies the whole syndrome of HE in all of its various forms. Ammonia can contribute to chronic low-grade glial edema [4] and alter the glutamate-glutamine metabolism during detoxification. In turn, glutamine may affect some astrocytic cytoskeletal components that not only determine morphology but also localize astrocytic functional proteins [5]. Ammonia can further potentiate the effects of neuro-inhibitors on the central nervous system [6], [7]. However, the considerable heterogeneity in etiology and disease severity increases the difficulty of establishing a gold standard for assessing the presence of HE. The role of ammonia also remains questionable.

Altered astrocyte morphology, chemical and electrical signaling within the brain networks, or even volume reduction in cirrhotic patients [8] may contribute to subsequent inappropriate cognitive performance and decline in consciousness. Aside from cerebral edema, network integrity assessment may help to directly delineate functional performance in HE. In particular, the default mode network (DMN) has been negatively associated with attention during the performance of demanding externally cued tasks. Completing the DMN also correlates well with the exhibition of consciousness [9]. Recently, DMN derived from coherent spontaneous blood oxygen level-dependent (BOLD) fluctuations in resting state functional MRI (rs-fMRI) has been extensively evaluated and thought to represent the neural consciousness stream [10]-[12]. Decreased DMN, especially of the posterior cingulate cortex (PCC), is associated with different degrees of impaired cognition and consciousness [13]–[15]. Without requiring demanding tasks [16], rs-fMRI is suited for investigating cognitive and consciousness disorders in liver cirrhosis.

The DMN is different between normal controls and HE patients [17]. However, network alteration in patients without HE and those with minimal HE is unknown. The gap between cerebral edema, ammonia concentration, and functional network is still unsolved. The aim of the present study was to investigate connectivity in resting-state networks correlating with consciousness across several HE levels under the hypothesis that increased cerebral edema evaluated by DTI is associated with a loss of connectivity in DMN, as assessed by rs-fMRI functional connectivity.

## Materials and Methods

### Subjects

Between August 2008 and December 2009, patients with liver cirrhosis were enrolled from Chang Gung Memorial Hospital, a tertiary referral centre. From the initial 51 patients, two patients with alcoholism-related cirrhosis were excluded and only patients with viral liver cirrhosis were included because alcohol addiction could alter brain function even without liver cirrhosis [18] and in order to unify the patho-physiology of HE. Three patients due to technical reasons, five without qualified MRI studies, and those with a history of drug abuse, psychiatric or neurologic illness, and head injury were likewise excluded. Forty-one patients were finally included for data analysis, including 30 men and 11 women (mean age 54.7 years; range, 26–70 years).

Cirrhosis was diagnosed based on clinical data and imaging studies [3]. The patients’ functional status was assessed using the Child-Pugh score [19], and 22 were classified as Child B and 19 as Child C. Overt HE (OHE) was graded using the West Haven criteria [20]. Patients with grade IV encephalopathy and those requiring sedation for MRI were excluded. Neurologic and neuro-psychological (NP) examinations were performed in all patients. Laboratory screening was done on the same day as the MRI scans and NP tests.

Thirty-two healthy volunteers (23 men and 9 women, mean age 54.3 years; range, 26–70 years) without a history of neurologic and psychiatric illnesses, unrelated medications, or head injury were recruited as healthy controls. All underwent detailed clinical and neurologic examinations in the same day as the MRI scans.

The hospital’s institutional review board human research committee approved the study. Because the study re-analyzed MRI data from a previous research in which written consent was already given by the patients for their information to be stored and used for research, the ethics committee waived the need for informed consent. All data were analyzed anonymously.

### Neuro-psychological Tests (NP Tests)

*General cognitive ability* was tested by the Cognitive Ability Screening Instrument (CASI) [21], *Executive dysfunction* by the Wisconsin Card Sorting Test (WCST) [22], and *Visuo-perceptive, motor and memory function* by the Taiwan Wechsler Adult Intelligence Scale III (Taiwan WAIS-III) sub-tests, including picture completion, digit-symbol, digit span, and block design [23].

Patients were grouped into “no HE”, “minimal HE” (mHE), and “OHE”. Eleven OHE patients were HE grade 1 (n = 3), grade 2 (n = 4), and grade 3 (n = 4) based on the West Haven criteria. Only three OHE patients completed the NP tests.

All 30 patients without overt HE completed the NP tests. According to Ferenci’s report [24], minimal HE was evaluated by the Wechsler Adult Intelligence Scale III (WAIS-III) subtests, including digit-symbol and block design [23]. An abnormality in at least one of these two tests was sufficient to define a patient as mHE. Based on the NP tests, 30 non-OHE patients were grouped as mHE (n = 14) and no HE (n = 16).

### Image Acquisition

The MR data were acquired on a 3.0T GE Signa MRI system (General Electric Healthcare, Milwaukee, WI, USA), while T1-weighted structural images were acquired using the 3D-FSPGR sequence (TR/TE 9.5/3.9 ms, flip angle 20°, field of view 24×24 cm, matrix size 512×512, 110 continuous slices with the slice thickness of 1.3 mm and in-plane spatial resolution of 0.47×0.47 mm).

#### DTI acquisition and analysis

The DTI were acquired using a single-shot echo-planar imaging sequence (TR/TE 15800/77 ms, number of excitation 3, matrix size 128×128, field of view 25.6 cm, voxel size 2×2×2.5 mm^3^, 55 axial oblique slices without gaps, 13 non-collinear directions with b-value 1000 s/mm^2^, and a non-diffusion weighted image volume with b-value 0 s/mm^2^). The total scanning time for each subject was 11 min and 35 sec.

Fractional anisotropy (FA) maps for each subject were computed using the in-house program and registered to the ICBM 152 template (Montreal Neurological Institute, MNI). The Talairach space was then approximated using the following steps [25]. First, non-diffusion weighted (b = 0) images of an individual subject were co-registered to their T1W images based on normalized mutual information as the cost function. The registration parameters were subsequently applied on the FA maps that were inherently registered to mean diffusivity (MD) images during the acquisition. These FA maps were also skull-stripped to remove non-brain tissue and background noise using the Brain Extraction Tool (BET) compiled in the FSL library 4.1 (Oxford Centre for Functional Magnetic Resonance Imaging of the Brain, Oxford University, Oxford, UK). Second, each T1W image was spatially normalized to the ICBM 152 template using the optimum 12-parameter affine transformation. Third, the co-registered FA maps were then transformed to the same stereotactic space as the T1W image by applying an affine transformation with 12 degrees of freedom together with a series of non-linear warps characterized by a linear combination of three dimension discrete cosine transform (DCT) basis functions. The estimated transformation parameters were also applied to rest-fMRI to register to the MNI space.

### Resting-state fMRI Acquisition and Analysis

“Resting state” fMRI scans were performed with the patients’ eyes closed using echo-planar imaging sequence (TR 2 s, TE 30 ms, FOV 240 mm, flip angle 80°, matrix size 64×64, thickness 4 mm, 300 scans of 32 contiguous axial slices) for a total scanning time of 10 min per subject. The first 10 scans of functional datasets were discarded to reach steady-state magnetization and allow participants to adapt to the scanning noise. Pre-processing steps included: (1) slice-timing correction for inter-leaved acquisition and head motion correction (<2.0 mm maximum displacement or 2.5° of any angular motion during the scanning); (2) data co-registration to the T1-weighted image of the same subject using an affine transformation; (3) normalization of co-registered functional images in the ICBM 152 template using the same transformation parameters as those in DTI post-processing, and further sub-sampling with a resolution of 2×2×2 mm^3^ and smoothened by a 6 mm full-width half maximum Gaussian kernel; and (4) linear de-trending to correct for signal drift and 0.01∼0.08 Hz band-pass filtering to reduce non-neuronal contributions to BOLD fluctuations [26], [27].

To ensure that each rs-fMRI dataset represented regionally specific neural activities, several sources of spurious variances were minimized by regressing out with white matter (WM), CSF, global signal, and six motion parameters [28]. All image pre-processing and data analysis were performed using the SPM8 (Wellcome Department of Cognitive Neurology, London, UK) in MATLAB 7.8.0 (MathWorks, MA) and REST software (http://www.restfmri.net).

#### Generation of seed regions of interest and calculation of functional connectivity

The pre-processed images were analyzed using seed-based connectivity analysis. The PCC was previously shown to be part of the DMN and reflected consciousness level [13]–[15]. The PCC seed described by Fox et al. [10], approximately spherical (volume 126 voxels, radius 6 mm) was used and located at (−5 −49 40) Talairach space that was converted to MNI space with coordinates (−5 −53 41). For each participant, this reference waveform was correlated with all other voxels to generate functional connectivity maps, while the r values were Fisher-transformed (z’) before further parametric statistical analyses to ensure that the data approximated a normal distribution.

#### Group level analyses

**Figure 1:**
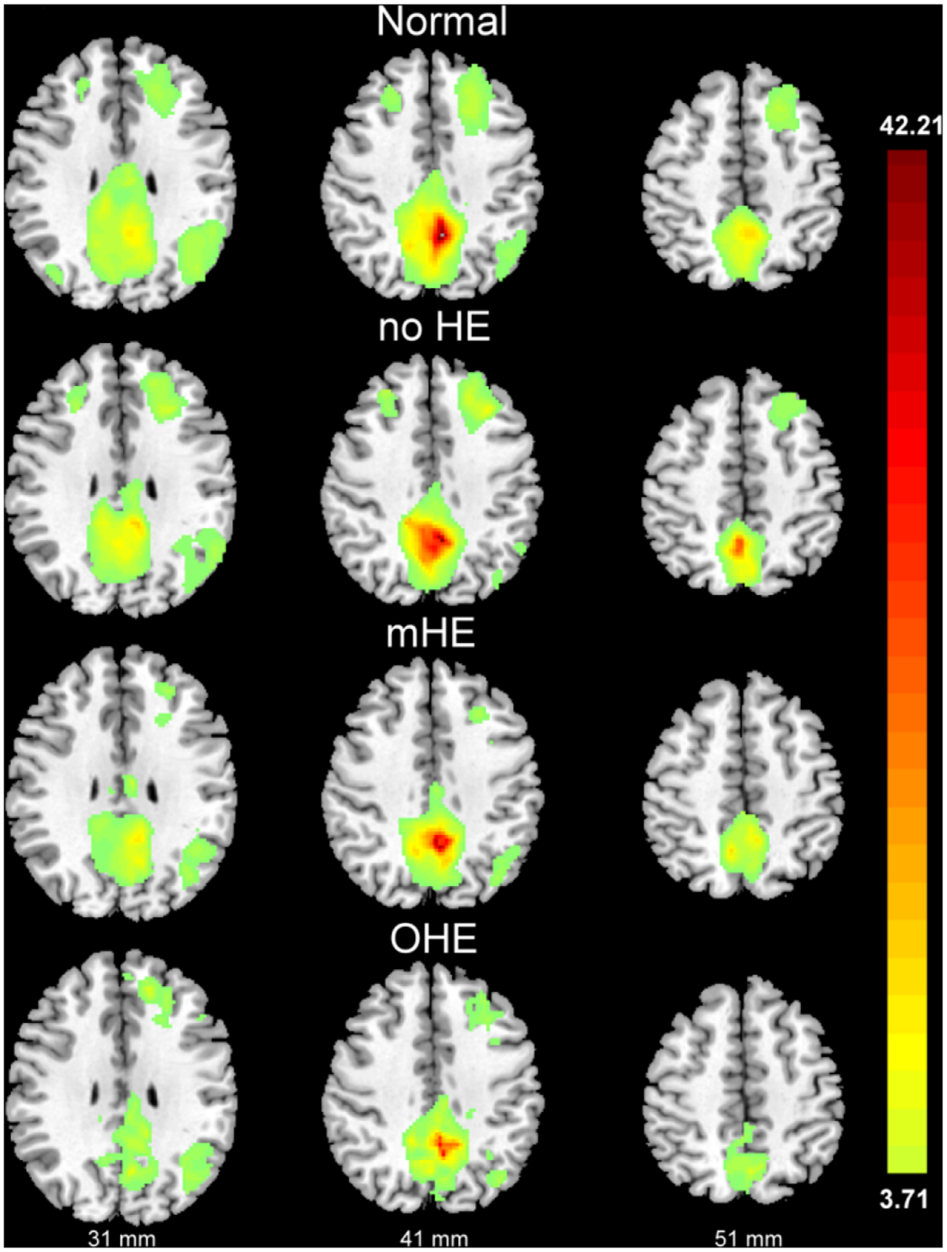
The fc-fMRI values relative to the PCC seed region. There were significant fc-fMRI values relative to the PCC seed region in the normal control, no hepatic encephalopathy (HE), minimal HE (mHE), and overt HE (OHE) groups at *p*<0.001 (corrected for multiple comparisons) using the mean T1-weighted structural images.

Group analyses were performed in SPM8 for the correlation maps of the PCC seed. A random effect one-sample (Fig. 1; Table 1) was calculated to compare the functional connectivity within groups (i.e., healthy control, no HE, mHE, and OHE) (*p*<0.001, FDR corrected, a voxel-wise threshold of p<10^−4^ was applied to the connectivity maps with a minimum cluster volume threshold of 536 mm^3^). In all subjects, the distribution of coherent PCC network activity was similar to that described in previous reports [29], [30]. Network activity included the seed region and its surroundings (PCC/precuneus and anterior cingulated cortex [ACC], middle frontal cortex [MFC], inferior parietal cortices, and angular gyri).

**Table 1:** Significant fc-fMRI values relative to the PCC seed region in normal control, no hepatic encephalopathy (HE), minimal HE (mHE), and overt HE (OHE) groups.

					Peak MNI coordinate		
Group	Seeds/Brain regions	BA	Peak T-value	Cluster Size	x	y	z
***Normal***	***PCC***						
	L Precuneus	7	42.21	10612	−4	−54	40
	L Middle frontal cortex	8	8.35	3941	−22	22	46
	L Angular cortex	39	7.33	2838	−44	−72	34
	R Middle frontal cortex	8	5.79	280	24	30	42
	R Angular cortex	39	5.19	95	38	−76	30
**No HE**	***PCC***						
	L Precuneus	7	34.51	3811	−2	−52	38
	L Middle frontal cortex	8	10.29	2447	−32	24	42
	L Middle temporal cortex	39	9.11	1581	−50	−68	20
	R Middle frontal cortex	8	7.61	326	28	36	40
**mHE**	***PCC***						
	L Precuneus	7	30.12	5494	−4	−50	40
	L Angular cortex	39	6.22	844	−42	−56	36
	L Middle frontal cortex	8	5.92	212	−24	22	40
**OHE**	***PCC***						
	L Precuneus	7	34.81	4436	−8	−50	40
	L Superior frontal cortex	9	10.36	657	−16	44	24
	L Angular cortex	39	8.01	686	−40	−72	38

Statistical threshold was set *p*<0.001, FDR corrected, voxel-wise threshold of *p*<10^−4^ was applied to the connectivity maps, with a minimum cluster size threshold of 67 = 576 mm^3^.

Abbreviations: BA, Brodmann area; PCC, posterior cingulate cortex; R, right; L, left.

#### Generation of ROIs

One-way analysis of variance (ANOVA) was used to compare the maps of four groups with age and sex as covariates. To avoid detecting clusters that did not appear in either groups’ maps, a masking procedure was used in which a group map for all subjects (significant clusters defined as in single-group analyses) was generated to limit the search for clusters that differed between the groups to this combined group map [31]. Significant clusters of activation were set at *p*<0.05, as determined by the AlphaSim program in the Analysis of Functional NeuroImages (AFNI) software [32]. Threshold was a combination of *p*<0.05 for voxel level and a minimum cluster size of 127 voxels with the mask file.

**Figure 2:**
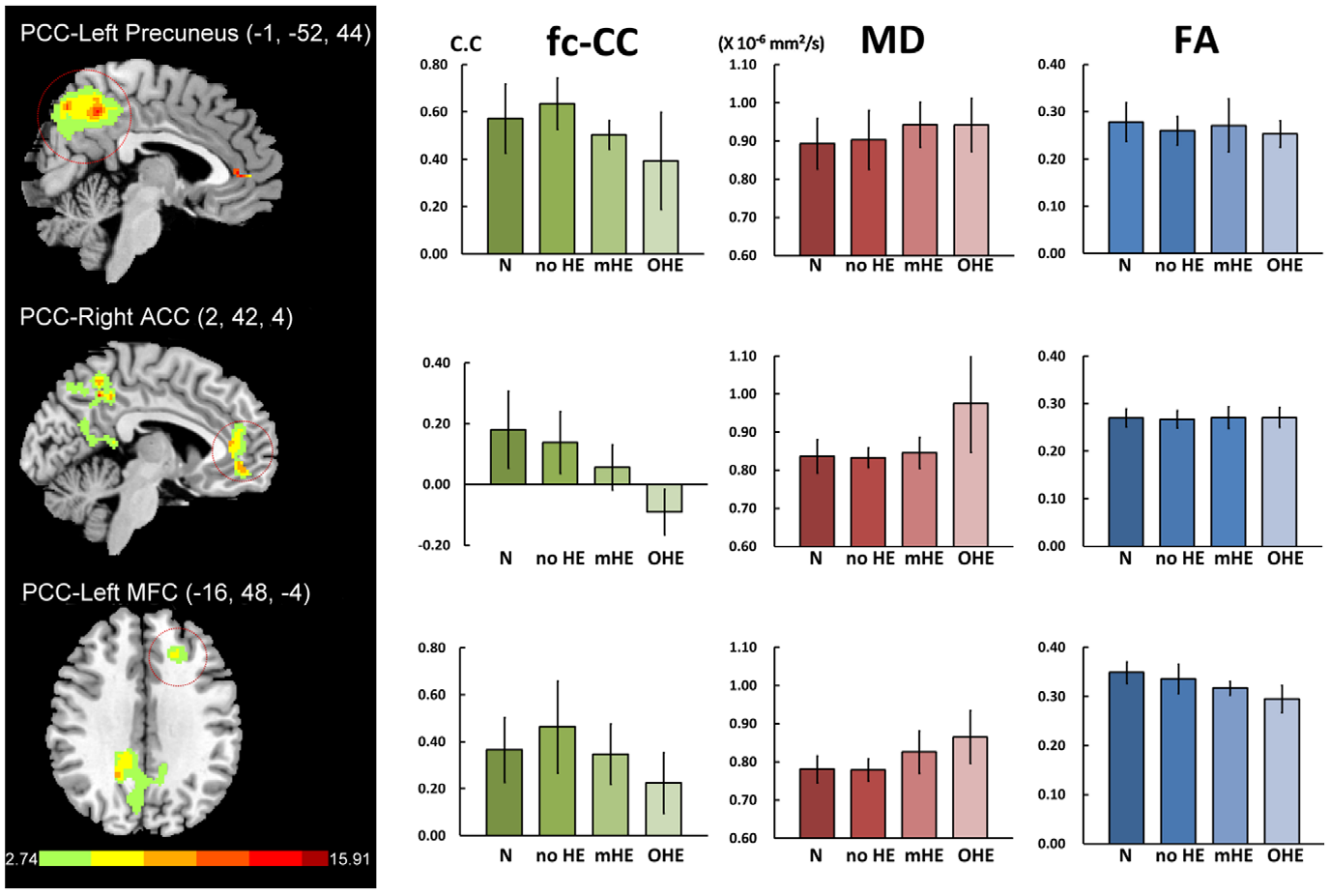
Differences of default model network between health subjects and liver cirrhosis and their corresponding MD and FA values. There were three biggest cluster areas in the PCC functional connectivity map, including the left precuneus, right ACC and left middle frontal cortex (MFC). MANCOVA revealed significant fc-CC [F = 4.415, *p* = 0.000] and MD [F = 3.944, *p* = 0.000] differences among the four groups, but not in FA [F = 0.859, *p* = 0.063].

The contrast of functional connectivity maps among groups exhibited the three biggest cluster areas in the PCC functional connectivity map, including the left precuneus, right ACC and left MFC (Fig. 2; Table 2). To examine differences among each patient group and healthy controls, the correlation coefficient of function connectivity (fc-CC) in these three ROIs was extracted. To evaluate the relationship with fc-CC, the corresponding MD and FA values were calculated within three previous ROI masks identified as the difference among group comparison procedures.

**Table 2:** Regions showing differences in fc-fMRI between normal subjects and cirrhotic patients in the PCC functional connectivity map.

					Peak MNI coordinate		
Seeds	Brain regions	BA	Peak F-value	Cluster Size	x	y	z
***PCC***	(ANOVA)						
	Left Precuneus	7	15.91	3058	−1	−52	44
	Right Anterior cingulate cortex	32	12.78	581	2	42	4
	Left Middle frontal cortex	10	6.49	139	−16	48	−4

The corrected statistical threshold was set at *p*<0.05, as determined by the AlphaSim program in Analysis of Functional NeuroImages (AFNI) software. Threshold was a combination of *p*<0.05 for a voxel level and a minimum cluster size of 127 voxels with the mask file. BA, Brodmann area.

### Statistical Analysis

Demographic data and clinical variables among healthy subjects, no HE, mHE, and OHE groups were analyzed using ANOVA. The NP results among groups were analyzed by analysis of co-variance (ANCOVA) with age, sex, and educational level as covariates. Comparison of fc-CC, MD, and FA in each ROI among groups were assessed using multiple analyses of variance (MANCOVA) with age and sex as covariates. Post-hoc univariate tests with Bonferroni’s correction were performed to follow-up significant main effects yielded by MANCOVA. Partial correlation test was used to examine the relationship between fc-CC, MD values in the three ROIs, clinical laboratory data, and CASI test, regressing out covariates of age and sex (and education for CASI tests). Statistical significance was defined at *p*<0.05. All analyses were done using the SPSS, Version 17 (SPSS Inc., Chicago, IL, USA).

## Results

### Progressive fc-CC Reduction and MD Elevation in Chronic Liver Disease with HE

The demographic features and NP tests were presented in Table 3. MANCOVA revealed significant fc-CC differences among the four groups (F = 4.415; *p*<0.001) (Fig. 2, Table 4). In the PCC map, decreased fc-CC in the left precuneus and right ACC was associated with increased HE severity. In the mHE groups, PCC exhibited decreased connectivity with the right ACC compared to the normal groups.

The MD values were derived from ROIs of the left precuneus, right ACC and left MFC in the PCC functional connectivity map. Using MANCOVA (Fig. 2, Table 4), the main effect of MD value had significant groups difference (F = 3.994, *p*<0.000). Follow-up one-way ANCOVAs revealed significant differences between overt HE and the other groups. There was also a trend of increased MD values from normal controls and no ME to the mHE group, but without significant difference among each other. There were also no significant FA differences among the groups in all ROIs.

**Table 3:** Demographics, clinical characteristics and neuro-psychiatric tests of liver cirrhosis patients and healthy controls.

	Control	No HE	mHE	OHE	F or X^2^	*p* value
# of subjects	32	16	14	11		
Age (years)	54.31 ± 10.34	50.13 ± 9.19	56.79 ± 4.68	58.73 ± 7.11	2.472	0.069
Gender	9F/23M	2F/14M	5F/9M	4F/7M	2.725	0.436
Education (years)	12.10 ± 4.18	10.86 ± 3.51	8.90 ± 3.54	6.00 ± 4.24	**5.745**	0.002*
Creatinine (mg/dL)	–	0.74 ± 0.36	0.78 ± 0.18	1.01 ± 1.07	0.759	0.475
GOT (IU/L)	–	64.13 ± 49.79	83.71 ± 23.39	413.1 ± 970.39	1.729	0.192
Bilirubin (mg/dL)	–	1.81 ± 1.41	2.83 ± 2.49	15.45 ± 17.40	8.480	0.001*
Venous ammonia (mg/dL)	–	120.38 ± 52.76	126.71 ± 68.74	158.18 ± 107.67	0.874	0.426
Albumin (mg/dL)	–	3.44 ± 0.64	3.06 ± 0.65	2.92 ± 0.38	2.802	0.074
International Normalized Ratio (INR)	–	1.17 ± 0.13	1.29 ± 0.27	3.20 ± 4.16	3.181	0.053
**General cognitive ability**						
CASI	93.28 ± 5.32	82.96 ± 14.60	80.37 ± 13.45	48.23 ± 29.74 (8)	**6.445**	0.001*
**Executive function**						
Perseverative response	11.27 ± 4.79	12.19 ± 2.35	17.28 ± 11.33	17.00 ± 4.36 (3)	**3.522**	**0.022**
Perseverative errors	10.90 ± 4.65	11.76 ± 2.41	13.42 ± 8.90	15.00 ± 3.60 (3)	0.684	0.566
Non-perseverative errors	14.53 ± 9.81	9.86 ± 4.64	22.14 ± 13.14	15.67 ± 19.40 (3)	1.378	0.261
Conceptual level response	48.02 ± 24.06	52.16 ± 23.96	25.89 ± 22.73	39.58 ± 28.40 (3)	1.908	0.141
Number of categories completed	2.33 ± 1.52	2.16 ± 1.48	0.71 ± 0.76	1.67 ± 1.53 (3)	2.508	0.070
**Visuo-perceptive, motor and memory function**				–		
Picture completion#	14.73 ± 5.37	13.77 ± 5.21	9.75 ± 5.60	–	2.103	0.113
Digit-symbol	67.00 ± 22.50	52.56 ± 22.94	41.86 ± 24.59	–	**3.492**	0.023*
Digit span	21.90 ± 4.61	17.52 ± 5.94	16.28 ± 5.79	–	**2.824**	0.049*
Block design	39.47 ± 13.52	36.88 ± 12.60	31.25 ± 6.87	–	**4.656**	0.007*

Results were mean±standard deviation.

Abbreviations: mHE, minimal hepatic encephalopathy; OHE, overt hepatic encephalopathy; CASI, Cognitive Ability Screening Instrument.

Statistical.

Threshold was set at *p*<0.05 (Boldface).

**Table 4:** Functional connectivity correlation coefficient, mean diffusivity and fractional anisotropy of healthy controls and patients with liver cirrhosis.

	N	A	B	C			
Mean ROI	Health, n = 32	no HE, n = 16	mHE, n = 14	OHE, n = 11	MANCOVA		Post hoc comparison
**Functional connectivity correlation coefficient** (z-value) F(4.415) *p* = 0.000							
PCC-Left Precuneus	0.572 (0.146)	0.635 (0.109)	0.503 (0.205)	0.393 (0.061)	**4.608**	0.006*	**C<A**
PCC-Right ACC	0.180 (0.127)	0.138 (0.102)	0.056 (0.075)	−0.091 (0.074)	**11.101**	0.000*	**C<N; C<A; C<B; B<N**
PCC-Left MFC	0.366 (0.138)	0.463 (0.196)	0.225 (0.129)	0.347 (0.129)	2.815	0.057	
**Mean diffusivity** (×10^−3^ mm^2^/s) F(3.944) *p* = 0.000							
PCC-Left Precuneus	0.893 (0.066)	0.903 (0.077)	0.943 (0.059)	0.942 (0.070)	0.545	0.654	
PCC-Right ACC	0.837 (0.044)	0.833 (0.026)	0.846 (0.041)	0.976 (0.129)	**8.689**	0.000*	**C<N; C<A; C<B**
PCC- Left MFC	0.781 (0.035)	0.779 (0.029)	0.826 (0.055)	0.865 (0.069)	5.288	0.003*	**C<N; C<A**
**Fractional anisotropy** F(1.859) *p* = 0.063							
PCC-Left Precuneus	0.278 (0.041)	0.260 (0.030)	0.271 (0.056)	0.253 (0.028)	0.459	0.712	
PCC-Right ACC	0.270 (0.019)	0.267 (0.018)	0.271 (0.023)	0.271 (0.021)	0.295	0.829	
PCC- Left MFC	0.349 (0.022)	0.336 (0.030)	0.317 (0.014)	0.295 (0.028)	6.044	0.001	

Abbreviations: ACC, anterior cingulate cortex; MFC, middle frontal cortex.

Statistical threshold was set at *p*<0.05 (Boldface).

### Association of fc-CC and Diffusion Indices in DMN

**Figure 3:**
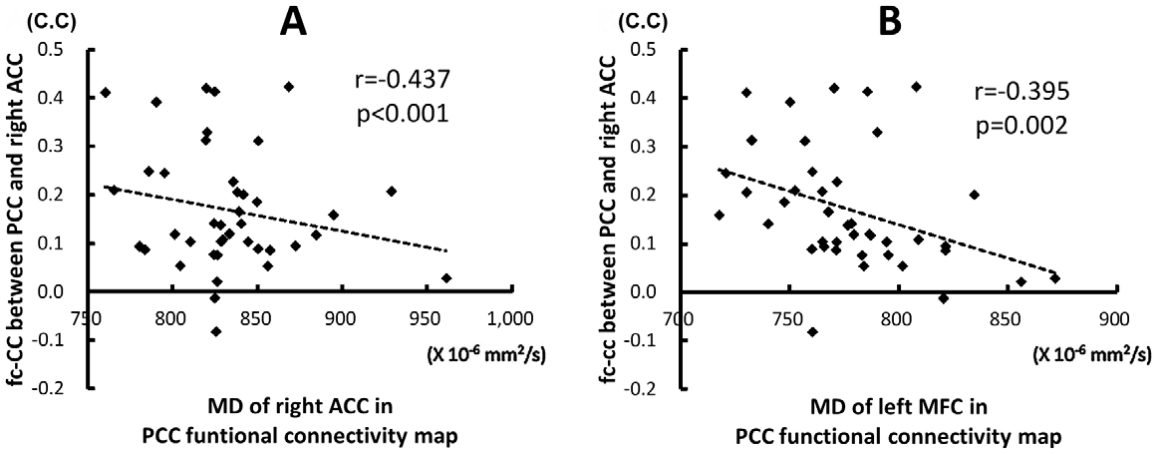
The relationship between fc-CC strength of the PCC-ACC and MD value. The decrease connectivity between PCC and ACC were associated with increase MD values derived from ROIs of the right ACC (A) and left middle frontal cortex (B) in the PCC functional connectivity map. C.C, correlation coefficient.

Increased MD value in the right ACC inversely correlated with decreased fc-CC strength between PCC and the right ACC (r = −0.437, *p*<0.000). Increased MD value in the left MFC also inversely correlated with decreased fc-CC strength between PCC and the right ACC (r = −0.395, *p* = 0.002) (Fig. 3).

### Correlation between fc-CC of DMN and Laboratory and Cognitive Tests

There was no significant correlation between the three ROIs - DMN fc-CC and the laboratory data. The decreased strength of inter-cortical fc-CC in the PCC-left Precuneus (r = 0.367, *p* = 0.006) and in the PCC-right ACC (r = 0.489, *p*<0.000) positively correlated with low CASI scores. Moreover, decreased fc-CCs in the PCC-left Precuneus and PCC-right ACC were also associated with lower CASI sub-tests (Table 5).

**Table 5:** Correlation between strength of inter-cortical fc-CC and cognitive variables after adjustments for age, gender and education.

fc-CC in ROIs	Clinical variable	Correlation (r)	*p* value
PCC-Left Precuneus	CASI total score	0.367	0.006
	Mental control	0.403	0.002
	Orientation	0.464	0.000
	Drawing	0.377	0.005
	Verbal fluency	0.305	0.023
PCC-Right ACC	CASI total score	0.489	0.000
	Mental control	0.421	0.001
	Attention	0.332	0.013
	Orientation	0.478	0.000
	Short term memory	0.481	0.000
	Abstraction	0.423	0.001
	Drawing	0.327	0.015
	Verbal fluency	0.404	0.002

Abbreviations: CASI, Cognitive Abilities Screening Instrument; fc-CC, functional connectivity correlation coefficient; PCC, posterior cingulate cortex; ACC, anterior cingulate cortex.

## Discussion

The BOLD signal from rs-fMRI is not a direct measure of neuronal activity but reflects local variations in de-oxyhemoglobin concentration from a combination of blood flow, blood volume, and oxygen metabolism [29]. The results are consistent with a previous PET study with impaired blood flow and oxygen metabolism in the frontal cortices and anterior cingulate gyrus in liver cirrhosis [33]. Changes in BOLD signal have been linked to synaptic activity of glutamate and its recycling through astrocytes from evidence on cell biology [34]. By using rs-fMRI, disconnection of BOLD signal coherence was proved to correlate with the disease progression.

Decreased fc-CC derived from the BOLD signal between inter-cortical regions may cause the underlying desynchronized neural activity of glutamate. Glutamate is the major metabolism of ammonia detoxification. However, we did not find significant association between venous ammonia level and functional connectivity in this study. Intervention for HE by lessening the ammonia concentration might alter results interpretation since ammonia level decrease can precede consciousness recovery. The altered glutamine-glutamate cycle can mediate many vital processes, including information encoding, memory formation and retrieval, spatial recognition, and consciousness maintenance [35]. Further animal studies should be conducted to validate these findings.

The main neuro-pathologic findings in HE is astrocyte swelling and intra-cranial hypertension [2]. Using DTI, interstitial edema is shown in HE *in vivo* [3]. Altering glutamate re-uptake during ammonia metabolism may result in the intracellular depletion of myo-inositol [36], an organic osmolyte that can trigger macromolecule migration to the extracellular space, resulting in increased extracellular fluid accumulation. This accounts for the increased MD value [3].

Another explanation for the elevated MD value may be the increased interstitial space from cell loss [37], [38]. The persistence of modified extra- and intra-cellular glutamate concentrations also alters the surrounding glial processes, while the associated mitochondrial dysfunction with oxidative stress may represent possible apoptosis with subsequent change in the brain network. However, the subtle FA decrease in the present study may only partially explain the hypothesis of cell loss.

The correlation between brain edema and network changes in HE has been documented by imaging modalities. However, the causal-relationship between morphology and function in cell level has not been demonstrated. In an animal study, hyper-ammonemia results in increased numbers of swollen astrocytes, increased immuno-reactivity of glutamine synthetase (GS), and some cytoskeletal proteins like the intermediate filament glial fibrillary acidic protein (GFAP) [39]. These increases in swollen astrocytes and GFAP immuno-reactivity can be reduced by GS inhibition [39].

The GFAP is expressed in astrocytes and is involved in cell structure maintenance, cell communication, and functioning of the blood brain barrier. It is also proposed to play a role in astrocyte-neuron interactions, which may explain the alteration of astrocyte morphology and function [40]. However, the limited correlation between decreased DMN integrity and ammonia level in this and another small sample-sized studies [17] also imply a more complicated process in HE development or an adaptive change of functional network that exists in each individual subject with a diverse clinical profile.

Our results reveal significant antero-posterior functional disconnection in the PCC functional maps (Fig. 2). Similar results have been suggested in subjects with increasing depth of sleep [14], wherein the posterior areas (bilateral inferior parietal cortex and PCC) strengthen their connectivity, while connections between the frontal and posterior regions are lost.

Functional uncoupling between the PCC and ACC and MFC may impair the brain’s ability to integrate information [16], [29]. Results of the present study suggest that integrated DMN activity may reflect ongoing conscious mental activity. In addition, increased MD, especially in the ACC, has good correlation with the disruption of the antero-posterior functional network, which further supports the notion that the PCC/precuneus are the central core within the DMN [41].

Early hepatic encephalopathy is not identified by structural abnormalities. Instead, a comprehensive neurologic examination is usually required. Revealing the subtle functional alteration in “sub-clinical HE” is even more difficult. In an FDG-PET study, mHE is associated with decreased glucose uptake and blood flow in the ACC, medial frontal region, and precuneus [33]. These indicate that brain regions involved in controlling the “attention system” responsible for monitoring is less active in mHE patients than in normal subjects. The NP tests, especially the block design and digit-symbol tests, are attention-demanding. Taken together, these findings support the hypothesis that a part of DMN connectivity fluctuates as it is more closely related to the presence of cognitive processes [15] and can be altered earlier in liver cirrhosis.

This study has some limitations. In subject recruitment, significantly ill patients were excluded from MRI study for safety concerns. As such, the preservation of DMN in comatose patients is unknown. Moreover, cirrhotic patients with altered consciousness are difficult to hold motionless during MRI scanning. Since the sample size is small, a relative low threshold for head motion criteria in data analysis (2.0 mm in translation and 2.5° rotation) has been applied, which might affect the results. Further studies with larger sample populations are warranted to assess the effect of therapy on functional network. Further validation of this diagnostic value on mHE by rs-fMRI is also required.

In conclusion, HE patients show connectivity de-coupling between the fronto-posterior areas of DMN, which is associated with the degree of HE and brain oedema. Rs-fMRI can be used to investigate variability among patients with differing symptom profiles, including sub-clinical states. Resting-state functional MRI may provide relevant supplemental information for monitoring HE patients and serve as a new biomarker for clinical diagnosis. Future studies that correlate rs-fMRI connectivity and metabolic alteration by MR spectroscopy, and those that investigate small-world topologic analysis by graphic theory and reversibility in post-treatment cirrhotic patients are recommended.
